# Recurrent Inguinal Hernia Post Laparoscopic Repair: A Retrospective Single-Center Study in Qassim Region, Saudi Arabia

**DOI:** 10.7759/cureus.13682

**Published:** 2021-03-03

**Authors:** Bandar Saad Assakran, Adel Mefleh Widyan, Abdulaziz S Al-lihimy, Abdullatif A Aljabali, Maha A Al-Enizi, Fadiyah A.

**Affiliations:** 1 General Surgery, King Fahad Specialist Hospital, Buraidah, SAU; 2 Mathematics, College of Science, Qassim University, Buraidah, SAU; 3 Medicine, Qassim University, Buraidah, SAU

**Keywords:** recurrent inguinal hernia, laparoscopic repair, saudi arabia

## Abstract

Introduction

Inguinal hernia is the most common hernia among the abdominal wall hernias. This study aims to estimate the long-term recurrence rate and laparoscopy-related risk factors for inguinal hernia at King Fahad Specialist Hospital in Buraidah, Al Qassim region, Saudi Arabia.

Methods

A single-center retrospective study of all laparoscopic hernia repair patients admitted in the surgical department of King Fahad Specialist Hospital in Buraidah, Al Qassim region, Saudi Arabia from January 2016 to July 2020.

Results

A total of 64 patients were included in the present study. All patients were male with a mean age 42.27±15.79 years. Out of 64 patients, 71.9% were married and 11 (17.2%) were smokers. Most patients were found to be in the elective priority (89.1%) and the emergency cases were 10.1%. A total of 6.3% had a recurrent hernia and 93.7% had a primary hernia. After testing the association of hernia repair and the patient-related factors, it was observed that there is no significant association between recurrent hernia repair and the mean age (p=0.072), body mass index (BMI) (p=0.962), smoking (p=0.347), married patients (p=0.196), and diabetes (p=0.446).

Conclusion

A total of 6.3 % of patients developed a recurrent inguinal hernia after laparoscopic repair. In contrast to the literature, this study showed that patient-related risk factors were not statically significant among our patients. However, the reasons behind the recurrence rate tend to be multifactorial, including surgical, technical, hospital capability, and patients factors. Therefore, early recognization and management of these risk factors are essential to prevent further cases.

## Introduction

Inguinal hernia is the most common hernia among abdominal wall hernias [1,2]. Inguinal hernia can range from being asymptomatic to painful especially with coughing, exercise, or bowel movement [1,2].

Although inguinal hernia is not an emergency, early surgical intervention is called for as it can progress to serious complications such as incarceration leading to obstruction and strangulation [3,4].

Nevertheless, the major concern after the repair is a recurrence, as it is a more complex and demanding procedure. The incidence of recurrent hernia irrespective to approach type is about 13%, while the recurrence rate following the laparoscopic approach has been reported to be between 1% and 7.9% in the last two decades [5].

Recurrent hernia is associated with many risk factors such as seroma and hematoma formation between the mesh and abdominal wall, and wound infection [5]. Direct inguinal hernia has a greater recurrence rate than indirect inguinal hernia, and recurrent inguinal hernia has a high risk to reoccur again after operation compared to a primary inguinal hernia [6]. Other factors are family history, which is considered a significant predictor, as well as age below 65 years old, diabetes, smoking, obesity, connective tissue degradation, and steroid intake [6,7]. A major technical risk is suturing an inguinal hernia instead of mesh-repairing the hernia. Higher recurrence happens when using short-term absorbable mesh fixation rather than long-term absorbable or nonabsorbable sutures, as well as laparoscopic hernia surgery compared to open hernia surgery. Also, local anesthesia carries a higher risk of recurrence compared with general anesthesia for primary hernia surgery [6,7].

The application of laparoscopic procedures has been increasing, this comes to no surprise as it has minimal postoperative pain, a shorter recovery period, and fewer infections compared to open repair [8]. The regular approach for hernia repair is mesh placement regardless of the type of operation [9].

There is insufficient recent data in Saudi Arabia on the recurrence rate after laparoscopically treated adult patients for inguinal hernia. Therefore, we aim to estimate the long-term recurrence rate and laparoscopy-related risk factors for inguinal hernia in Saudi Arabia.

## Materials and methods

This was a single-center retrospective study of all laparoscopic hernia repair patients admitted in the surgical department of King Fahad Specialist Hospital in Buraidah, Al Qassim region, Saudi Arabia from January 2016 to July 2020.

I. Source of data and sample size

All inguinal hernia patients who underwent laparoscopic hernia repair in our center during the study period and aged ≥18 years were included. Based on the prior studies [11-13] that were conducted in a single center to estimate the recurrence rate, the sample size range was estimated to be from 25-51. Therefore, our target was comparable to them.

II. Data collection

We reviewed the medical records of included patients retrospectively. Our collection sheet is divided into many sections including biographic data, history, physical exam, intra-operative notes, etc. Then, we divided our patients into two groups: with and without documented recurrent hernia. Recurrent hernia is defined as reappearing of hernia and failure of primary repair. Finally, the different measures mentioned in the collection sheet were used to compare between study groups. 

III. Statistical analysis

Patients' data was analyzed by using Statistical Package for Social Science (SPSS, IBM Corp., Armonk, NY) then summarized in two tables. In table one, baseline characteristics of patients were presented as frequencies, percentages, and mean ± standard deviations. The differences between patients and recurrent cases were tested using Chi-square test or t-test and a p-value < 0.05 was considered statistically significant.

IV. Ethical consideration

Ethical approval was obtained from King Fahad Specialist Hospital administration, and from the local Institutional Review Board (IRB) in Qassim region, Saudi Arabia vide approval number 1441-2157972.

## Results

A total of 64 patients were included in the present study. All patients were male with a mean age 42.27±15.79 years. Out of 64 patients, 71.9% were married, 11 (17.2%) were smokers. Most patients were found to be in the elective priority (89.1%), and the emergency cases were 10.1%. A total of 6.3% of patients had recurrent hernia and 93.7% had primary hernia as represented in Table 1.

**Table 1:** Baseline characteristics of patients HTN: hypertension; DM: diabetes mellitus; COPD: chronic obstructive pulmonary disease; ASA: American Society of Anesthesiologists physical status classification; LOS: length of stay

Variables		Mean±SD / n (%)
Sample Size		64
Age (in years)		42.27±15.79
Male Patients		64 (100%)
BMI (kg/m^2^)		26.15±4.11
Nationality (Saudi)		61 (95.3%)
Marital Status (Married)		46 (71.9%)
Smoking		11 (17.2%)
Co-existing Conditions	None	46 (71.9%)
	HTN	3 (4.7%)
	DM	3 (4.7%)
	Asthma	4 (6.3%)
	COPD	1 (1.6%)
	More than one	7 (10.9%)
Duration of Complain	< 1 Year	41 (64.1%)
	> 1 Year	23 (35.9%)
Side	Unilateral	55 (85.9%)
	Bilateral	9 (14.1%)
Priority	Emergency	7 (10.9%)
	Elective	57 (89.1%)
Hernia Repair	Primary	60 (93.7%)
	Recurrent	4 (6.3%)
ASA	I	30 (46.9%)
	II	34 (53.1%)
	III	0 (0.0%)
LOS (days)		2.14±1.74
Returning to work (days)		8.90±7.92

**Figure 1:**
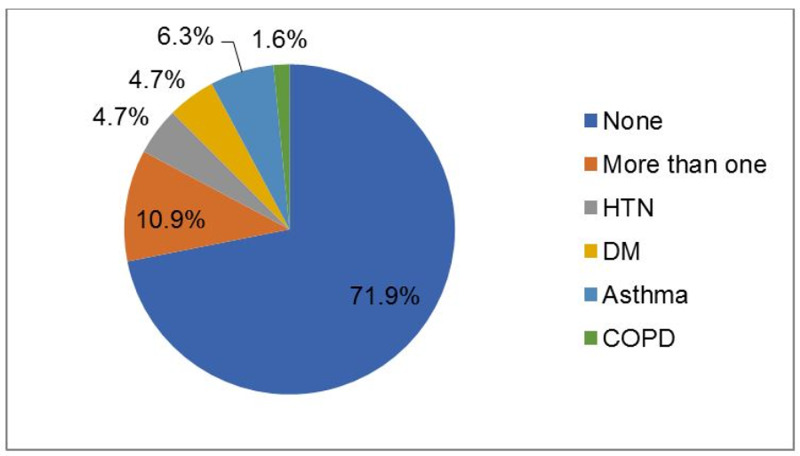
Co-existing conditions HTN: hypertension; COPD: chronic obstructive pulmonary disease; DM: diabetes mellitus

Most of the patients had had no co-existing conditions (71.9%), 6.3% had asthma, 4.7% had hypertension, and one patient (1.6%) had chronic obstructive pulmonary disease, as shown in Figure 1.

Table 2 shows the association and difference between patients who has primary versus recurrent hernia repair; Chi-square test and t-test were used. After testing the association of hernia repair and patient-related factors, it was observed that there is no significant association between recurrent hernia repair and the mean age (p=0.072), BMI (p=0.962), smoking (p=0.347), married patients (p=0.196), and diabetes (p=0.446).

**Table 2:** Association and difference between patients who had primary repair versus recurrent repair (Chi-square test and t-test) ASA: American Society of Anesthesiologists physical status classification; HTN: hypertension; DM: diabetes mellitus; COPD: chronic obstructive pulmonary disease; BPH: benign prostatic hyperplasia

Variables		Primary N=60	Recurrent N=4	P-Value	
Age		41.35±15.35	56.00±18.18	0.072	
BMI		26.15±4.02	26.05±6.19	0.962	
Smoking		11 (18.3%)	0 (0.0%)	0.347	
Marital Status (Married)		42 (70.0%)	4 (100.0%)	0.196	
Side	Unilateral	52 (86.7%)	3 (75.0%)	0.516	
	Bilateral	8 (13.3%)	1 (25.0%)		
ASA	I	28 (46.7%)	2 (50.0%)	0.897	
	II	32 (53.3%)	2 (50.0%)		
	III	0 (0.0%)	0 (0.0%)		
Duration of Complain	< 1 Year	38 (63.3%)	3 (75.0%)	0.545	
	> 1 Year	22 (36.7%)	1 (25.0%)		
Co-existing Conditions	None	43 (71.7%)	3 (75.0%)	0.446	
	More than one	7 (11.7%)	0 (0.0%)		
	HTN	2 (3.3%)	1 (25.0%)		
	DTM	3 (5.0%)	0 (0.0%)		
	Asthma	4 (6.7%)	0 (0.0%)		
	BPH	0 (0.0%)	0 (0.0%)		
	COPD	1 (1.7%)	0 (0.0%)		
	Constipation	0 (0.0%)	0 (0.0%)		

## Discussion

Our current study retrospectively follows 64 patients treated laparoscopically for inguinal hernia to investigate the association between specific risk factors and the occurrence of recurrent hernia. Inguinal hernia surgery has evolved as technology and materials have advanced. Yet, surgeons occasionally encounter a case of recurrent hernia in adult patients after primary repair. When it comes to the rate of the recurrence, the analysis of our data has shown that 6.3% had a recurrence while 93.7% had a primary occurrence of inguinal hernia. This is comparative to a study done by Koju et al., in which they compared the results of the laparoscopic trans-abdominal pre-peritoneal (TAPP) mesh repair versus the open Lichtenstein’s hernioplasty in 102 patients, each group containing 51 participants. The rate of recurrence was 5% in the TAPP group while in the Lichtenstein group was nil [10]. Langeveld et al. presented similar results as well in a research in which they compared laparoscopic totally extraperitoneal (TEP) repair with Lichtenstein’s open mesh repair in a randomized control trial. 336 of a total of 660 patients were randomized to TEP procedure, while 324 to Lichtenstein. After a mean follow-up of 49 months, the recurrence rate for the two procedures was 3.8% and 3.0%, respectively [11].

Alas, many studies describe inguinal hernia surgery for recurrence and re-recurrence as a complex and challenging procedure that requires a lot of experience. This is all to avoid intra-operative and post-operative complications. Scar tissue that formed after primary reoccurrence is one cause of intra-operative complications as it distorts the usual anatomical landmarks, such as the corona mortis, triangle of doom, and triangle of pain, which could lead to dissection of the nerves, injuring the spermatic cord, bladder, vas deferens, bowel, or the inferior epigastric vessel. Placing ticks on these landmarks could lead to bleeding or chronic pain as well. An important post-operative concern is re-recurrence, which could be a result of improper recurrence repair that could lead to the deterioration of the mechanical strength of the tissue and further distortion of anatomical landmarks, resulting in the vicious cycle of multiple recurrence repairs [12,13].

Based on what has been reported in the literature, recurrent inguinal hernia post laparoscopic repair might occur at any time post-operatively along with multiple contributing risk factors, this could include surgical, hospital, and patient-related factors [14]. Furthermore, Siddaiah-Subramanya et al. concluded that surgeon’s experience, inappropriate surgical technique, and inadequate orifice closure by unsuitable mesh size, all have an important role in increasing recurrent cases [5]. Another study done by Novik et al. reported that hospital capability is considered one of the major causes of recurrence [15]. The patient-related factors investigated in this study, such as age, BMI, diabetes, smoking, and marital status revealed not to be significantly associated with recurrent cases. A study conducted by Schjøth-Iversen et al. with a total of 1,047 patients revealed that BMI less than 30, and patient age were insignificant risk factors, this appears to be similar to our study [16]. Still, it comes in contrast to previous studies that suggest that smoking and diabetes result in a poor healing process, leading to a higher recurrence rate [16-18].

One of the limitations in our study is the lack of data on the reappearance of hernia post laparoscopic repair among married patients; therefore, the authors recommend that further studies investigate the recurrence rate among married cases. Lastly, missing factors other than that related to the patient, which were mentioned earlier, as well as the small sample size, might explain the reason behind the insignificant association amongst our patients.

## Conclusions

A total of 6.3% of the patients in our study developed a recurrent inguinal hernia after laparoscopic repair. In contrast to the literature, this study showed that patient-related risk factors were not statistically significant among our patients. However, the reasons behind the recurrence rate tend to be multifactorial, including surgical, technical, hospital capability, and patient-related factors. Therefore, early recognition and management of these risk factors are essential to prevent further cases.
